# A novel glycolysis-related gene signature for predicting the prognosis of multiple myeloma

**DOI:** 10.3389/fcell.2023.1198949

**Published:** 2023-06-02

**Authors:** Bingxin Zhang, Quanqiang Wang, Zhili Lin, Ziwei Zheng, Shujuan Zhou, Tianyu Zhang, Dong Zheng, Zixing Chen, Sisi Zheng, Yu Zhang, Xuanru Lin, Rujiao Dong, Jingjing Chen, Honglan Qian, Xudong Hu, Yan Zhuang, Qianying Zhang, Zhouxiang Jin, Songfu Jiang, Yongyong Ma

**Affiliations:** ^1^ Department of Hematology, The First Affiliated Hospital of Wenzhou Medical University, Wenzhou, Zhejiang, China; ^2^ Department of Hepatobiliary Surgery, The Second Affiliated Hospital and Yuying Children’s Hospital of Wenzhou Medical University, Wenzhou, Zhejiang, China; ^3^ Key Laboratory of Intelligent Treatment and Life Support for Critical Diseases of Zhejiang Province, Wenzhou, Zhejiang, China; ^4^ Zhejiang Engineering Research Center for Hospital Emergency and Process Digitization, Wenzhou, Zhejiang, China

**Keywords:** multiple myeloma, glycolysis, prognostic signature, tumor microenvironment, risk stratification, therapeutic targets

## Abstract

**Background:** Metabolic reprogramming is an important hallmark of cancer. Glycolysis provides the conditions on which multiple myeloma (MM) thrives. Due to MM’s great heterogeneity and incurability, risk assessment and treatment choices are still difficult.

**Method:** We constructed a glycolysis-related prognostic model by Least absolute shrinkage and selection operator (LASSO) Cox regression analysis. It was validated in two independent external cohorts, cell lines, and our clinical specimens. The model was also explored for its biological properties, immune microenvironment, and therapeutic response including immunotherapy. Finally, multiple metrics were combined to construct a nomogram to assist in personalized prediction of survival outcomes.

**Results:** A wide range of variants and heterogeneous expression profiles of glycolysis-related genes were observed in MM. The prognostic model behaved well in differentiating between populations with various prognoses and proved to be an independent prognostic factor. This prognostic signature closely coordinated with multiple malignant features such as high-risk clinical features, immune dysfunction, stem cell-like features, cancer-related pathways, which was associated with the survival outcomes of MM. In terms of treatment, the high-risk group showed resistance to conventional drugs such as bortezomib, doxorubicin and immunotherapy. The joint scores generated by the nomogram showed higher clinical benefit than other clinical indicators. The *in vitro* experiments with cell lines and clinical subjects further provided convincing evidence for our study.

**Conclusion:** We developed and validated the utility of the MM glycolysis-related prognostic model, which provides a new direction for prognosis assessment, treatment options for MM patients.

## 1 Introduction

Multiple myeloma (MM) is the second most common hematological malignancy and is attributed to bone marrow infiltration of monoclonal plasma cells (Laubach et al., 2011; Siegel et al., 2021). It is characterized by hypercalcemia, renal damage, anemia, and bone lesions (Röllig et al., 2015; Kumar et al., 2017). MM is a highly heterogenous disease, that exits on a continuous disease spectrum ranging from precancerous states monoclonal gammopathy of undetermined significance (MGUS) and smoldering multiple myeloma (SMM) driven by the accumulating genetic changes and immune escape (Kyle et al., 2002; Kyle et al., 2007; Landgren et al., 2009).

In the past decade, novel treatments have increased MM’s survival rates, such as proteasome inhibitor bortezomib (BTZ) and immunomodulatory drug (IMiD) thalidomide (Kumar et al., 2008; Bianchi et al., 2015; Moreau et al., 2015). Despite the improvements in overall survival, relapse frequently occurs (Krishnan et al., 2016; Wallington-Beddoe et al., 2018). Meanwhile, MM is still an incurable disease, and the prevalence continues to rise owing to the aging population (Cowan et al., 2018). The Revised International Staging System (R-ISS) combines cytogenetic abnormalities (CAs), serum lactate dehydrogenase (LDH), and ISS traits and is the most widely recognized risk-stratification tool for newly diagnosed multiple myeloma (NDMM) patients (Palumbo et al., 2015). However, a large number of people with heterogeneous risk factors were concentrated in R-ISS stage II (Cho et al., 2017; Jung et al., 2018). New approaches are needed to better risk stratify MM patients to inform prognosis and treatment options for better individualized management.

The most crucial hallmark of cancer is metabolic reprogramming (Hanahan, 2022). Cancer cells preferentially employ glycolysis, even when there is abundant oxygen. This trait of cancer is known as “Warburg effect” (Warburg, 1956). Consistently, a typical feature of MM is the presence of an increased glycolytic gene profile and sensitivity to glycolysis inhibitors (D'Souza and Bhattacharya, 2019). Several key enzymes in glycolysis were highly upregulated in MM, including hexokinase 2 (HK2) and lactate dehydrogenase A (LDHA) (He et al., 2015; Ikeda et al., 2020). Furthermore, compared with NDMM patients, these enzymes were upregulated more significantly in relapsed and bortezomib-refractory MM patients (Mulligan et al., 2007; Maiso et al., 2015). Drugs targeting glycolysis have increasingly become a promising direction for cancer treatment. HK2 inhibitor, 3-bromopyruvate (3-BP), in addition to disrupting ATP production, significantly impaired autophagy and induced apoptosis, which was demonstrated in MM cells (Ikeda et al., 2020). Likewise, the GLUT4 inhibitor ritonavir has been shown to reduce the proliferation and viability of myeloma and improve chemotherapy sensitivity (McBrayer et al., 2012). Dichloroacetic acid is thought to achieve the apoptosis and proliferation inhibition of MM cells by activating the pyruvate dehydrogenase complex, and can increase the sensitivity of MM cell lines to bortezomib (Sanchez et al., 2013). Given the significance of glucose metabolism reprogramming, it is crucial to investigate the prognostic signatures and novel treatments of MM from the perspective of glycolysis.

In this study, we constructed a risk scoring model based on glycolysis-related genes (GRGs) to predict the prognosis and guide clinical treatment of MM. We further performed therapeutic response prediction and comparative analyses of biological function and tumor microenvironment (TME) to explore the underlying mechanisms. Then, a nomogram combing the gene signature and clinical manifestations was developed to improve the predictive power and clinical applicability. Finally, the expression of the selected genes was further verified by quantitative real-time PCR (qRT-PCR).

## 2 Materials and methods

### 2.1 Data collection

From the Gene Expression Omnibus (GEO) database (http://www.ncbi.nlm.nih.gov/geo/), expression profiles were downloaded for 4 MM datasets, including GSE136337, GSE24080, GSE4204, and GSE6477. The datasets were log2 transformed after normalising with a microarray muite 5.0 (MAS5.0) method. Detailed clinicopathological data and survival data were also extracted from GEO (Table 1). The GSE136337 was used as the training, while the GSE24080 and GSE4204 datasets were used for validation. And we used the GSE6477 to reveal the evolution of GRGs in normal individuals, MGUS, SMM, and active MM groups. Glycolysis-related genomes “c2.cp.biocarta.v7.2.symbols”, “c2.cp.hallmark.v5.0.symbols”, “c2.cp.reactome.v7.5.symbols”, “c2.cp.kegg.v7.2.symbols”, “c2.cp.wikipathways.v7.2.symbols” in Gene Set Enrichment Analysis (GSEA) (http://www.gsea-msigdb.org/gsea/msigdb) were the source of the GRGs. After taking intersections with the three GEO datasets (GEO136337, GSE24080 and GSE4204), 293 genes were finally used for further study (Supplementary Table S1).

**TABLE 1:** Clinical traits of the training and validation cohorts.

Characteristics	Training cohort	Validation cohort	Validation cohort
	GSE136337	GSE24080	GSE4204
	(*n* = 415)	(*n* = 556)	(*n* = 534)
Sex			
Female	158 (38%)	222 (40%)	—
Male	257 (62%)	334 (60%)	—
Age			
≤65 years	297 (72%)	421 (76%)	—
>65 years	118 (28%)	135 (24%)	—
Albumin			
≥3.5 g/dL	331 (80%)	481 (87%)	—
<3.5 g/dL	84 (20%)	75 (13%)	—
β2M			
<3.5 mg/L	187 (45%)	320 (58%)	—
3.5–5.5 mg/L	109 (26%)	118 (21%)	—
≥5.5 mg/L	119 (29%)	118 (21%)	—
LDH			
≤250 U/L	392 (94%)	507 (91%)	—
>250 U/L	23 (6%)	49 (9%)	—
Del (17p)			
False	400 (96%)	—	—
True	15 (4%)	—	—
t (4,14)			
False	401 (97%)	—	—
True	14 (3%)	—	—
t (14,16)			
False	414 (99%)	—	—
True	1 (1%)	—	—
ISS			
I	163 (39%)	296 (53%)	—
II	133 (32%)	142 (26%)	—
III	119 (29%)	118 (21%)	—
R-ISS			
I	149 (36%)	—	—
II	243 (59%)	—	—
III	65 (16%)	—	—
Risk score			
High	206 (50%)	278 (50%)	267 (50%)
Low	207 (50%)	278 (50%)	267 (50%)
Survival			
Alive	239 (58%)	386 (69%)	442 (83%)

### 2.2 Construction and validation of a glycolytic prognostic model

The prognostic glycolytic genes identified by univariable Cox regression analysis (*p* < 0.001) were then subjected to the Least absolute shrinkage and selection operator (LASSO) Cox regression analysis. Using the R package “glmnet”, the penalty parameter *λ* was set as 0.09 to determine the best weighting coefficient of glycolytic genes.

### 2.3 Interaction network and genetic variation map of glycolysis-related genes

The co-expression correlation matrix of the 12 genes was constructed by “ggcorrplot” R package. The protein-protein interaction (PPI) network of the 12 genes was acquired from the STRING database (version 11.5) (https://www.string-db.org/) (Szklarczyk et al., 2011). The genetic mutation landscape of the GRGs in MM was explored with The Cancer Genome Atlas (TCGA) (https://portal.gdc.cancer.gov/) with the “maftools” package and cBioPortal for Cancer Genomics (http://www.cbioportal.org/). We also used the Cancer Cell Line Encyclopedia database (CCLE, https://portals.broadinstitute.org/ccle) to further validate these prognostic genes’ expression.

### 2.4 Comparative analysis of clinical characteristics and treatment responsiveness between subgroups

To further investigate the heterogeneity between subgroups, we compared clinical characteristics and drug sensitivity among the subtypes. As for cytogenetics, based on the existing data in the training dataset and the previous findings, we defined high-risk cytogenetic abnormalities (HRCAs) as follows: del17p, amp1q, t (4; 14), t (14; 20), t (14; 16) or MYC aberrations determined by fluorescent *in situ* hybridization (FISH) or conventional cytogenetics (Chng et al., 2014; Sonneveld et al., 2016; Abdallah et al., 2020; Schmidt et al., 2021; Wallington-Beddoe and Mynott, 2021). Patients with at least one HRCA were defined as the high-risk cohort. The low-risk subgroup included those with other abnormalities [del13q, del16q, del1p, del1q, t (11; 14), t (12; 14)]. The rest belonged to the non-mutation cohort.

The “pRRophetic” package was used to assess the drug susceptibility between the low- and high-scoring group (Geeleher et al., 2014a; Geeleher et al., 2014b). This method builds the statistical model based on the data from the Cancer Genome Project (CGP) (Garnett et al., 2012), consisting of baseline gene expression microarray data and sensitivity to 138 drugs in a panel of almost 700 cell lines.

### 2.5 Exploration of biological functions based on prognostic glycolytic signature

To reveal the underlying mechanism, we employed the weighted gene co-expression network analysis (WGCNA) (Langfelder and Horvath, 2008, 2012). Also, we conducted an association study between modules and clinical aspects and identified critical genes linked to the model. Further use of the Metascape platform (https://metascape.org/gp/index.html) was made to implement the Gene Ontology (GO) analysis of the important genes. GSEA (GSEA v4.2.2 software, http://software.broadinstitute.org/gsea/login.jsp) was also conducted to uncover the differences in biological functions with the Kyoto Encyclopedia of Genes and Genomes (KEGG). Statistical significance was defined as *p* < 0.05 and q < 0.25.

### 2.6 Characterization of the TME and immune treatment responsiveness of the glycolytic model

We employed five techniques to measure the immune microenvironment of subgroups, including EPIC, MCPCounter (Becht et al., 2016), quanTIseq (Plattner et al., 2020), the single-sample gene set enrichment analysis (ssGSEA) and xCell (Aran, 2020), to remove variances across different algorithms. The correlation analysis of the GRGs with immune-related genes and functional status from Thorsson et al. was performed. (Thorsson et al., 2018). mRNAsi is a tool for evaluating how closely cancer cells resemble stem cells (Malta et al., 2018). The sensitivity to immune checkpoint inhibitor (ICI) was evaluated using the immunophenotype score (IPS) (Charoentong et al., 2017) and the tumor immune dysfunction and exclusion (TIDE) (Jiang et al., 2018).

### 2.7 Constructing a predictive nomogram to assess clinical applicability

A nomogram combing age, ISS stage and glycolytic risk score was constructed. For the nomogram’s self-verification, the calibration curve was conducted. Based on the merged scores and other clinical parameters, time-dependent receiver operating characteristic (time-ROC) curves were computed for 1-, 3-, and 5-year survival. With the “ggDCA” package, survival net benefits of each clinical feature and the risk score were estimated with decision curve analysis (DCA). DCA curves focus on evaluating the clinical benefit of the model, predicting which threshold probability range treatment for patients in the model will achieve a higher clinical benefit, meeting the practical needs of clinical decision-making. For the prediction model, the net benefit (NB) is a composite indicator that incorporates both true positives and false positives. In the DCA curve, there are two reference lines, one with no intervention for anyone, called treat_none, and the other with intervention for everyone, called treat_all. The model has real value only if its NB is higher than both treat_all and treat_none at some threshold probability (Vickers and Elkin, 2006).

### 2.8 Cell lines and cell culture

MM cell lines including LP-1, RPMI8226, NCI-H929, MM1.R, I9.2, and U266 cells were collected from Fenghui Biotechnology Co., Ltd. (Hunan, China). Cells were grown in an incubator set at 37 °C with a humid environment that contained 5% CO2. In addition to 10% fetal bovine serum, the cells were cultured in RPMI-1640 medium (Gibco, Shanghai, China) with 100 IU/mL penicillin and 100 mg/mL streptomycin.

### 2.9 Patients

35 MM patients were subjected to our experiment, of whom four were relapsed MM (RMM). As controls, normal bone marrow samples were taken from 21 healthy volunteers for PCR on cell lines and patient samples. Table 2 displayed the clinical traits of the patients. The study was authorized by the First Affiliated Hospital of Wenzhou Medical University’s ethics committee, and all operations were conducted according to the informed consent and Helsinki Declaration.

**TABLE 2:** The clinical information of the subjects in the experimental validation.

Variables	Levels	MM (*n* = 35)	Normal (*n* = 21)	*P*
		NRMM (*n* = 31) RMM (*n* = 4)		
Sex	Female	12 (39%) 1 (25%)	8 (38%)	0.610
	Male	19 (61%) 3 (75%)	13 (62%)	—
Age (years)	<65	9 (29%) 2 (50%)	9 (43%)	0.672
	≥65	22 (71%) 2 (50%)	12 (57%)	—
Isotype	IgG	14 (45%) 2 (50%)	—	—
	IgA	10 (32%) 0 (0%)	—	—
	IgD	1 (3%) 0 (0%)	—	—
	Light chain	6 (20%) 2 (50%)	—	—
Albumin (g/dL)	≥3.5	16 (52%) 3 (75%)	—	—
	<3.5	15 48%) 1 (25%)	—	—
β2M (mg/L)	<3.5	13 (42%) 2 (50%)	—	—
	3.5–5.5	6 (19%) 2 (50%)	—	—
	≥5.5	12 (39%) 0 (0%)	—	—
LDH (U/L)	≤250	25 (81%) 3 (75%)	—	—
	>250	6 (19%) 1 (25%)	—	—
Del (17p)	False	31 (100%) 4 (100%)	—	—
	True	0 (0%) 0 (0%)	—	—
IgH rearrangement	False	30 (97%) 4 (100%)	—	—
	True	1 (3%) 0 (0%)	—	—
Del (13q)	False	22 (74%) 4 (100%)	—	—
	True	9 (26%) 0 (0%)	—	—
Amp1q	False	22 (71%) 3 (75%)	—	—
	True	9 (29%) 1 (25%)	—	—
ISS	I	5 (16%) 1 (25%)	—	—
	II	14 (45%) 3 (75%)	—	—
	III	12 (39%) 0 (0%)	—	—
R-ISS	I	5 (16%) 1 (25%)	—	—
	II	24 (77%) 3 (75%)	—	—
	III	2 (7%) 0 (0%)	—	—
Myeloma cells (%)	<10	9 (29%) 2 (50%)	—	—
	≥10	22 (71%) 2 (50%)	—	—
Calcium (mmol/L)	≤2.65	31 (100%) 3 (75%)	—	—
	>2.65	0 (0%) 1 (25%)	—	—
Serum creatinine	<177	24 (77%) 4 (100%)	—	—
(μmol/L)	≥177	7 (23%) 0 (0%)	—	—
Hb (g/L)	≥85	20 (65%) 1 (25%)	—	—
	<85	11 (35%) 3 (75%)	—	—
Bone lesions	0	13 (32%) 1 (25%)	—	—
	1–3	2 (6%) 0 (0%)	—	—
	>3	16 (52%) 3 (75%)	—	—

NRMM, non-relapsed multiple myeloma; RMM, relapsed multiple myeloma.

### 2.10 Quantitative real-time PCR

Using the Righton DNA&RNA Blood and Tissue Kit (Righton Bio, Shanghai, China), total RNA was isolated from total bone marrow-derived mononuclear cells as directed by the manufacturer. The cDNA synthesis kit (Vazyme, Nanjing, China) was used to execute reverse transcription in order to produce cDNAs. With the help of the Taq Pro universal SYBR qPCR Master Mix (Vazyme, Nanjing, China), qRT-PCR was used to find the levels of glycolysis-related gene expression, with *β-ACTIN* acting as an internal reference. The primers used was provided in Table 3.

**TABLE 3:** Primers used in the study.

Gene symbol	Polarity	Sequence 5′-3′
*LDHB*	forward	CCT​CAG​ATC​GTC​AAG​TAC​AGT​CC
	reverse	ATC​ACG​CGG​TGT​TTG​GGT​AAT
*MDH2*	forward	GCC​ATG​ATC​TGC​GTC​ATT​GC
	reverse	CCG​AAG​ATT​TTG​TTG​GGG​TTG​T
*MPC2*	forward	GCT​CCG​TTG​TGA​GGA​GAG​AC
	reverse	GCT​CTG​CAC​TCC​ACA​CTG​AA
*PRPS1*	forward	ATC​TTC​TCC​GGT​CCT​GCT​ATT
	reverse	TGG​TGA​CTA​CTA​CTG​CCT​CAA​A
*PAXIP1*	forward	ACA​ATG​CAC​TAG​CCT​CAC​ACA
	reverse	ACA​CTG​AAC​GGA​CAG​AAT​CAC
*POLR3K*	forward	CAC​CCG​CAA​GGT​AAC​AAA​TCG
	reverse	CTG​CAT​GAA​GTA​AGC​ACG​AGG
*SOD1*	forward	GGT​GGG​CCA​AAG​GAT​GAA​GAG
	reverse	CCA​CAA​GCC​AAA​CGA​CTT​CC
*NSDHL*	forward	CAA​GTC​GCA​CGG​ACT​CAT​TTG
	reverse	ACT​GTG​CAT​CTC​TTG​GCC​TG
*AURKA*	forward	GGA​ATA​TGC​ACC​ACT​TGG​AAC​A
	reverse	TAA​GAC​AGG​GCA​TTT​GCC​AAT
*CYB5A*	forward	CAC​CAC​AAG​GTG​TAC​GAT​TTG​A
	reverse	CAT​CTG​TAG​AGT​GCC​CGA​CAT
*PAM*	forward	CTG​GGG​TTA​CAC​CTA​AAC​AGT​C
	reverse	GCT​TGA​AGT​CAA​TCA​CGA​AGG​C
*GMPPB*	forward	GGG​AAT​CCG​AAT​CTC​CAT​GTC
	reverse	GTC​TCA​GAG​AGT​AGG​TCA​CGG
*β-ACTIN*	forward	TCA​AGA​TCA​TTG​CTC​CTC​CTG​AG
	reverse	ACA​TCT​GCT​GGA​AGG​TGG​ACA

### 2.11 Statistical analyses

The statistical analysis was performed by SPSS version 24.0 (SPSS Inc., Chicago, IL, United States), R software version 4.1.1 (R Foundation for Statistical Computing, Vienna, Austria), and GraphPad Prism version 9.0.0 (GraphPad-Software Inc., San Diego, CA, United States). The Student’s t test was used to compare two groups for quantitative variables with a normal distribution. The Chi-square test was for the categorical variables. The Mann-Whitney *U* test was employed for the abnormal distributional variables. Multiple groups were compared using one-way analysis of variance (ANOVA) and the Kruskal-Wallis test. LSD test is used for multiple comparisons after ANOVA. Pearson’s correlation test was for the correlation evaluation between variables with normal distributions, and the Spearman’s correlation test was for variables with aberrant distributions. Statistics were judged significant at *p* < 0.05.

## 3 Results

### 3.1 Sample selection and clinicopathological features

The glycolytic model was developed using the GSE136337. The GSE24080 and GSE4204 were used for model validation. Survival data were available for 1,514 subjects in the three datasets (GSE136337, *n* = 424; GSE24080, *n* = 556; GSE4204, *n* = 534). Clinicopathological features in sufficient samples allowed for subsequent Cox regression analysis (GSE136337, *n* = 415; GSE24080, *n* = 556) (Table 1). Moreover, the GSE6477 (*n* = 162) was used to reveal the evolution of GRGs in normal individuals, MGUS, SMM, and active MM groups.

### 3.2 Construction and validation of a glycolytic model

**FIGURE 1:**
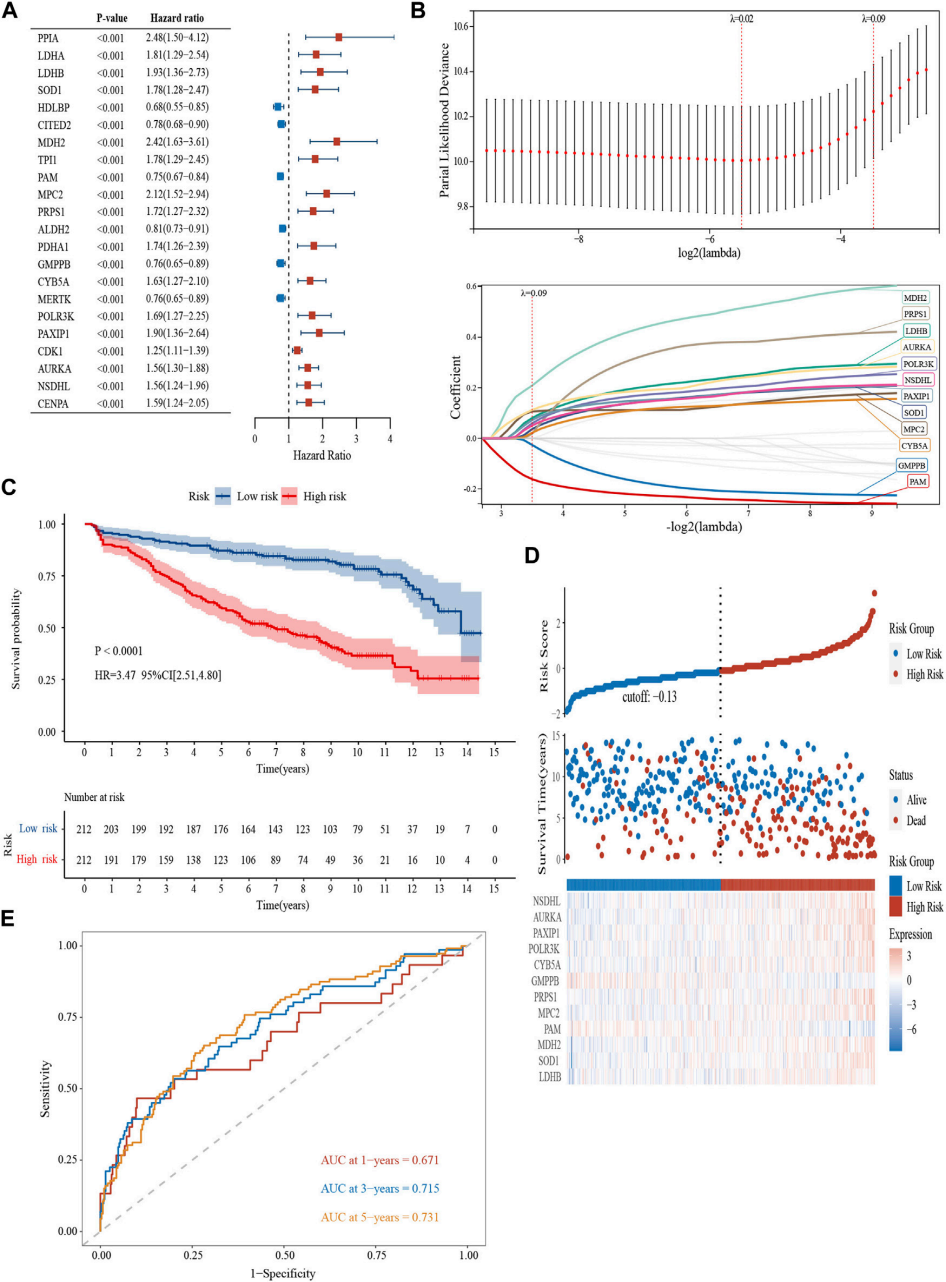
Construction and validation of a glycolytic prognostic model (GSE136337, *n* = 424). **(A)** Forest plot of hazard ratios manifesting the prognostic values of glycolysis-related genes. **(B)** LASSO Cox regression analysis for variable selection. **(C)** Kaplan-Meier curves of patients in the high- and low-risk group (*p* < 0.0001). **(D)** The AUC of the model assessed by time-dependent ROC curves. **(E)** The distribution of the survival outcomes and the expression of the prognostic genes among the subgroups.

In the GSE136337 (*n* = 424), 22 genes related to glycolysis were associated with prognosis (*p* < 0.001) (Figure 1A). Then, the glycolytic model was constructed using 12 genes (*λ* = 0.09) (Figure 1B). The formula was as follows: glycolytic risk score = (0.0803 × *LDHB*) + (0.0365 × *SOD1*) + (0.2087 × *MDH2*) - (0.1617 × *PAM*) + (0.1063 × *MPC2*) + (0.0182 × *PRPS1*) - (0.0262 × *GMPPB*) + (0.0217 × *CYB5A*) + (0.0593 × *POLR3K*) + (0.0721 × *PAXIP1*) + (0.1125 × *AURKA*) + (0.0507 × *NSDHL*). The median risk score was used to divide the subjects into high-risk and low-risk subgroups.

Kaplan–Meier curves revealed survival differences among the subtype in the training (Figure 1C) and the two validation datasets (Supplementary Figures S1A, B). The high-scoring population had worse survival (HR = 3.47, 95% CI = 2.51–4.80, *p* < 0.0001; HR = 2.46, 95% CI = 1.78–3.41, *p* < 0.0001; HR = 2.09, 95% CI = 1.35–3.22, *p* < 0.001). The heterogeneity of survival outcomes was further validated in Figure 1D; Supplementary Figures S1C, D. Heat maps were also plotted to compare the expression of the 12 glycolytic genes. In general, the high-risk groups had lower levels of *GMPPB* and *PAM* expression, whereas other genes exhibit the opposite pattern (Figure 1D; Supplementary Figures S1C, D). AUCs of the 1-, 3-, and 5-year survival were 0.671, 0.715, and 0.731 in the GSE136337 (*n* = 424) to assess the sensitivity and specificity (Figure 1E). The results of the validation datasets were exhibited in Supplementary Figure S1E (GSE24080, *n* = 556) and 1F (GSE4204, *n* = 534).

### 3.3 Interaction network and genetic variation map of glycolysis-related genes

**FIGURE 2:**
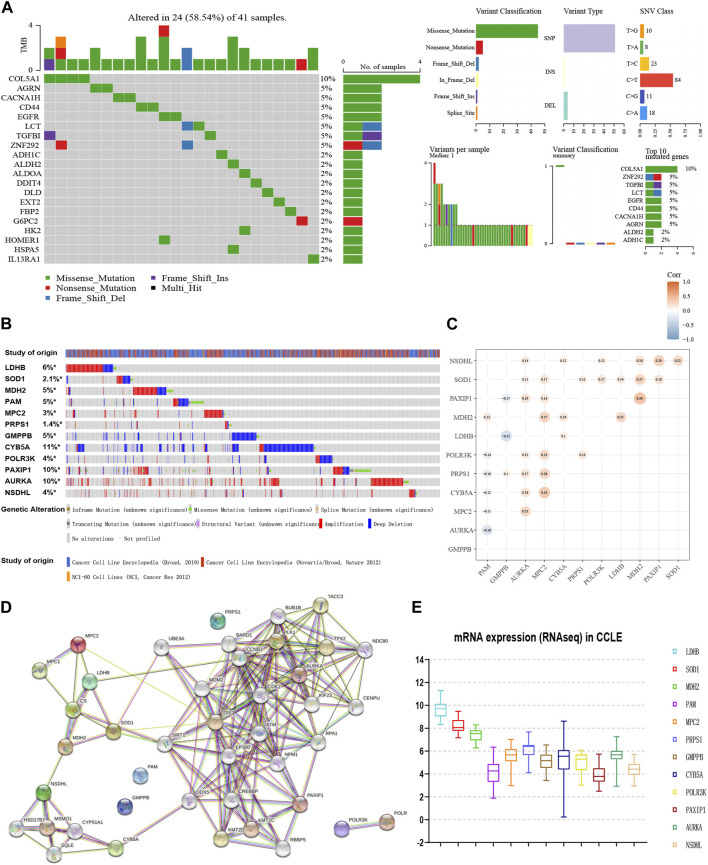
Interaction network and genetic variation map of glycolysis-related genes. **(A)** The genetic mutation landscape of the candidate genes from TCGA-MM. **(B)** The SNVs and CNVs of the 12 genes with cBioPortal for Cancer Genomics. **(C)** Co-expression analyses of 12 glycolytic gene signatures (GSE136337, *n* = 424). **(D)** Protein-protein interaction network of the 12 genes and other closely related proteins. **(E)** The external validation of the expression levels of the 12 genes using CCLE.

Depending on the TCGA data, we explored the mutation landscape of 293 genes in the candidate glycolytic gene set in MM. Figure 2A showed the top 20 genes with the highest mutation rate. Among them, missense mutations accounted for the major part and the single nucleotide polymorphisms (SNPs) occurred more frequently than insertions or deletions. Furthermore, we used cBioPortal to reveal the single nucleotide variants (SNVs) and copy number variations (CNVs) of the 12 genes (Figure 2B). The alterations of *LDHB*, *SOD1*, *MDH2*, *MPC2*, *PRPS1*, *AURKA*, *PAXIP1* and *NSDHL* in cancer cell lines were 6%, 2.1%, 5%, 3%, 1.4%, 10%, 10%, and 4% separately. Amplification was the most common change. In contrast, both *PAM* and *GMPPB* were altered at a frequency of 5%, and their most common change was deletion (Figure 2B). These results were in general agreement with our data.

Moreover, the co-expression matrix and the PPI network showed a close relationship between the 12 genes (Figures 2C, D). In the CCLE database, *LDHB*, *SOD1*, *MDH2*, *MPC2*, *PRPS1*, *CYB5A*, *POLR3K*, and *AURKA* were over-expressed at the cellular level in MM, while *PAM* and *GMPPB* were under-expressed (Figure 2E), corresponding to the model equation above.

### 3.4 Comparative analysis of clinical characteristics and treatment responsiveness between subgroups

**FIGURE 3:**
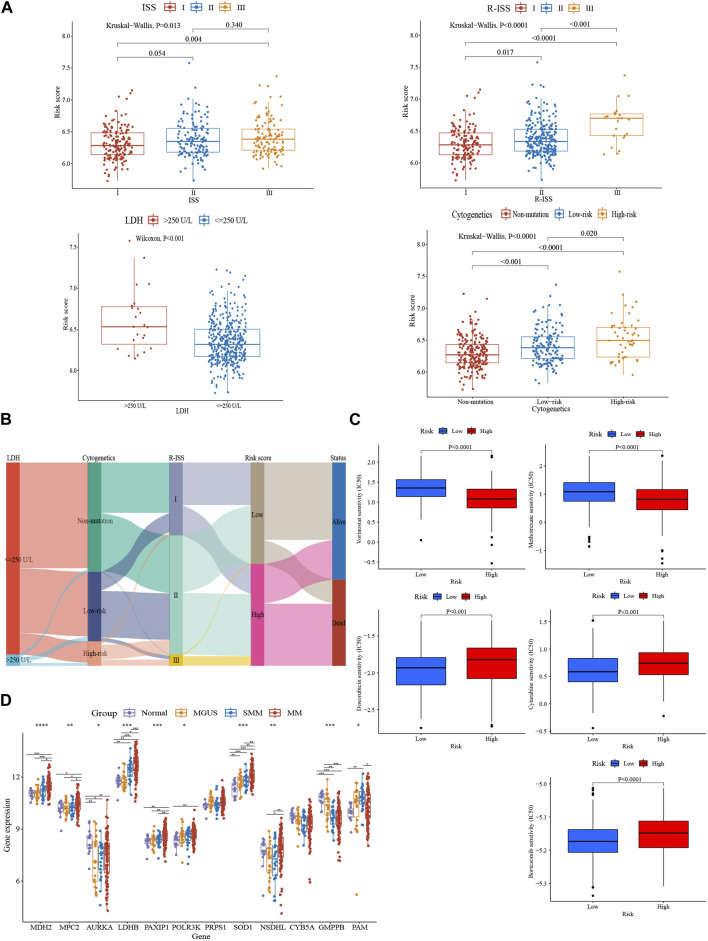
Comparative analysis of clinical characteristics and treatment responsiveness between subgroups. **(A)** Relationship between risk score and distinct clinical traits (GSE136337, *n* = 415). **(B)** A sankey diagram exhibiting the changes of LDH level, R-ISS stage, cytogenetic variation risk, glycolytic risk score, and prognosis (GSE136337, *n* = 415). **(C)** Evaluations of the drug susceptibility among the subtypes (GSE136337, *n* = 424). **(D)** Variations in these 12 genes’ expression levels as MM progressed (GSE6477, *n* = 162). *p < 0.05; **p < 0.01; ***p < 0.001; ****p < 0.0001.

The glycolytic risk score was shown to be independently associated with survival (GSE136337, *n* = 415, HR = 3.191 (2.295, 4.436), *p* < 0.001; GSE24080, *n* = 556, HR = 1.662 (1.214, 2.275), *p* = 0.020) (Table 4). Subsequently, we analyzed the relationship between the 12-gene signature and clinicopathological traits in the GSE136337 (*n* = 415). The risk scores of the higher LDH and the high-risk cytogenetics groups were higher than those counterparts (*p* < 0.001) (Figure 3A). We observed the gradually increasing tendency of the risk score with the increasing ISS or R-ISS stage (*p* < 0.05) (Figure 3A). The distribution of the patient’s characteristics and survival outcomes were illustrated in Figure 3B.

**TABLE 4:** Univariate and multivariate Cox regression analyses of survival in the training and validation cohorts.

Characteristics	Training cohort GSE136337 (*n* = 415)				Validation cohort GSE24080 (*n* = 556)			
	Univariate analysis		Multivariate analysis		Univariate analysis		Multivariate analysis	
	Regression coefficient (SE)	*P*	Hazard ratio (95% CI)	*P*	Regression coefficient (SE)	*P*	Hazard ratio (95% CI)	*P*
Age (<65 vs. ≥65 years)	0.579 (0.155)	<0.001	1.596 (1.715–2.168)	0.003	0.174 (0.177)	0.327	—	—
Sex (female vs. male)	−0.248 (0.154)	0.107	—	—	−0.052 (0.156)	0.739	—	—
Albumin (≥3.5 vs. <3.5 g/dL)	0.410 (0.177)	0.021	—	—	0.595 (0.194)	0.002	—	—
β2m (<3.5 vs. 3.5–5.5 vs. ≥5.5 mg/L)	0.469 (0.091)	<0.001	—	—	0.512 (0.088)	<0.001	—	—
LDH (≤250 vs. >250 U/L)	0.732 (0.270)	0.007	—	—	1.316 (0.197)	<0.001	—	—
del (17p)	0.098 (0.417)	0.814	—	—	—	—	—	—
t (4,14)	0.035 (0.455)	0.939	—	—	—	—	—	—
t (14,16)	0.719 (1.003)	0.474	—	—	—	—	—	—
CA (non-mutation vs. low-risk vs. high-risk)	0.346 (0.102)	<0.001	—	—	—	—	—	—
ISS (Ⅰ vs. Ⅱ vs. Ⅲ)	0.503 (0.095)	<0.001	1.549 (1.282–1.872)	<0.001	0.526 (0.090)	<0.001	1.562 (1.304–1.870)	<0.001
R−ISS (Ⅰ vs. Ⅱ vs. Ⅲ)	0.595 (0.133)	<0.001	—	—	—	—	—	—
Risk (low vs. high)	0.618 (0.155)	<0.001	3.191 (2.295–4.436)	<0.001	0.627 (0.159)	<0.001	2.160 (1.555–2.999)	<0.001

Albumin, β2M, and LDH were not included in the multivariate analysis, because of co-linearity with the ISS or R-ISS.

CA, cytogenetics alteration.

According to the estimated IC50 values of the chemotherapeutic agents, the high-risk cohort showed the resistance to bortezomib, doxorubicin, and cytarabine (*p* < 0.001), but more sensitive to methotrexate and vorinostat (*p* < 0.0001), compared to the low-risk group (Figure 3C). Additionally, glycolysis-related gene expression changes were also present in the evolving disease spectrum of myeloma (GSE6477, *n* = 162) (Figure 3D). The expression levels of *LDHB* and *SOD1* exhibited an increasing trend as the disease progressed. In addition, *MDH2*, *MPC2*, *PAXIP1,* and *NSDHL* were upregulated in myeloma patients. *GMPPB*, however, revealed a pattern of decline.

### 3.5 Exploration of biological functions based on prognostic glycolytic signature

**FIGURE 4:**
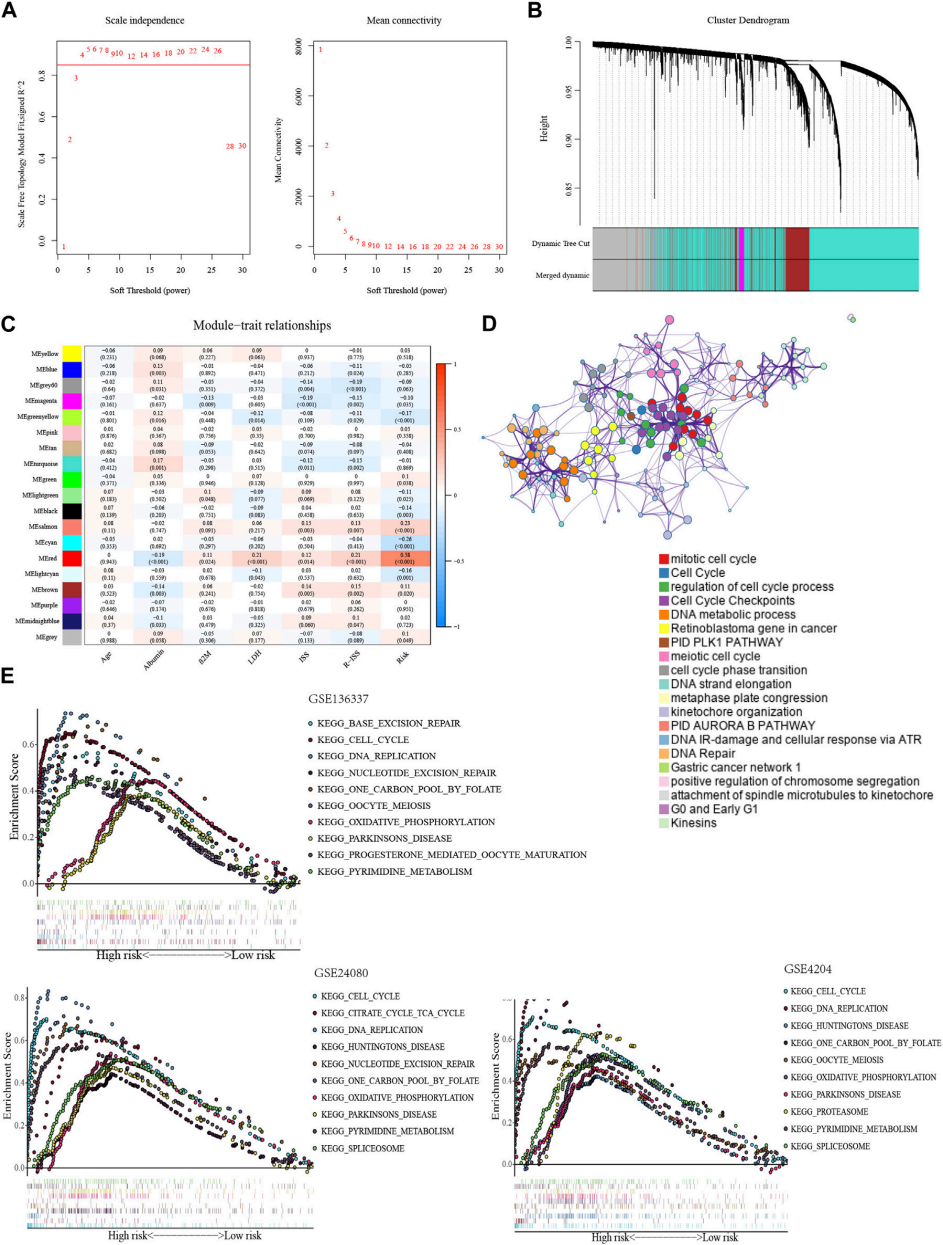
Exploration of biological functions based on the glycolytic signature. **(A)** Selection of optimal soft thresholds in WGCNA (GSE136337, *n* = 424). **(B)** Hierarchical clustering dendrogram of genes using WGCNA (GSE136337, *n* = 424). **(C)** Associations of gene module with risk model and clinical features (GSE136337, *n* = 415). **(D)** Enrichment clustering network using Metascape database (GSE136337, *n* = 424). **(E)** The top 10 enriched pathways in the training dataset and validation datasets (GSE136337, *n* = 424; GSE24080, *n* = 556; GSE4204, *n* = 534).

We conducted an investigation of biological functions among subgroups to uncover the mechanism underlying the glycolytic signature. WGCNA was implemented to build a weighted gene co-expression network. To ensure proper connectivity and sustain the network nearly scale-free, the soft threshold was set at four (Figure 4A). 19 gene modules were produced after grouping related modules (Figure 4B). The red module and the risk score had the strongest association (r = 0.58, *p* < 0.001) (Figure 4C). The red module’s 221 genes were analyzed for GO enrichment using the online database Metascape. Important processes in tumors, including the mitotic cell cycle, cell cycle checkpoints, DNA metabolism, and the retinoblastoma gene in cancer, were the main sites of gene module concentration (Figure 4D).

In GSEA, the high-scoring population was home to the considerably enriched pathways, which were mostly linked to glycolysis and included pyrimidine metabolism, one carbon pool via folate, and oxidative phosphorylation. Consistent with previous results, the pathways of cell cycle, DNA repair, and nucleotide excision repair can also be found (Figure 4E).

### 3.6 Characterization of the TME and immune treatment responsiveness of the glycolytic model

**FIGURE 5:**
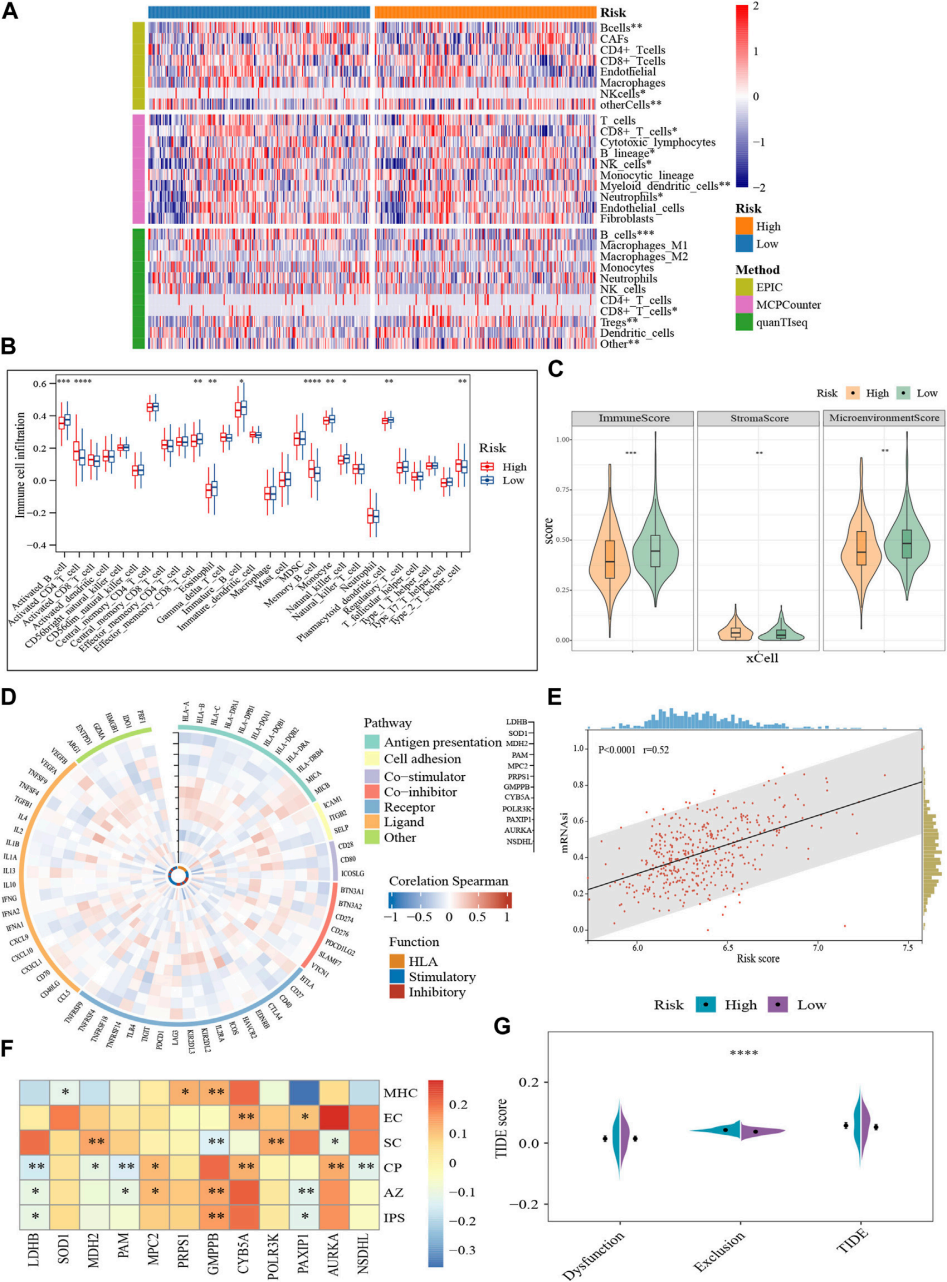
Characterization of the tumor microenvironment and immune treatment sensitivity of the glycolytic model (GSE136337, *n* = 424). **(A)** Visualization of differences in immune cell abundance based on the various algorithms. **(B)** Comparison of 28 immune cell infiltration levels between high- and low-scoring cohorts **(C)**. Immune-related scores calculated by xCell. **(D)** Correlation of prognostic genes with immune-related pathways and functions. The vertical axes with black arrows indicate different prognostic genes. **(E)** Correlation between risk score and stemness index. **(F)** Relationships of prognostic genes with distinct immune phenotypes. **(G)** Assessment of T-cell function and infiltration levels. IPS, immunophenotype score; MHC, antigen presentation; EC, effector cells; SC, suppressor cells; CP, checkpoint marker; z-score, AZ; TIDE, tumor immune dysfunction and exclusion. *p < 0.05; **p < 0.01; ***p < 0.001; ****p < 0.0001.

The immune microenvironment is incredibly important to the growth of MM. Based on several different algorithms, the heat map demonstrated the heterogeneity in the immune landscape among the subgroups (Figure 5A). Immune cell infiltration levels were higher in the low-scoring cohort (*p* < 0.05), such as active B cells, effector memory CD8^+^ T cells, immature B cells, NK cells, plasmacytoid dendritic cells, and Th2, while stromal cell abundance was higher in the high-scoring population (*p* < 0.01) (Figures 5B, C). Moreover, we performed a correlation analysis of the GRGs with immune-related genes and functional status. The expression of most prognostic genes was negatively correlated with the immune-related genes (Figure 5D). For example, *LDHB*, *SOD1*, *MDH2*, *POLR3K*, and *PAXIP1* were negatively correlated with immune-related genes in terms of antigen presentation and cell adhesion, while *GMPPB* was positively correlated (Figure 5D).

Meanwhile, higher risk scores were observed to be strongly associated with the stemness index (Figure 5E). IPS characterizes the immune phenotype of the cells from four perspectives (“antigen-presenting, AP; effector cells, EC; suppressor cells, SC; checkpoints, CP”) (Charoentong et al., 2017). And a total score (z-score, AZ) is finally generated after normalization. A higher z-score corresponds to higher ICI responsiveness. Consistent to our previous results, *GMPPB* was positively correlated with antigen presentations (*p* < 0.01). Compared to *GMPPB*, *MDH2,* and *POLR3K* were linked to higher concentrations of immunosuppressive cells (*p* < 0.01). Furthermore, high expression of *GMPPB* was often accompanied by greater sensitivity to ICI (*p* < 0.01), while *LDHB* and *PAXIP1* showed the opposite pattern (*p* < 0.05) (Figure 5F). The quantity and functional status of T cells is important mediators in immune-targeted therapy. The T-cell exclusion was more prone to occur in the TME of the high-scoring cohort (*p* < 0.0001), foreshadowing their possible resistance to ICI (Figure 5G). In conclusion, glycolysis-related markers may be a useful tool for determining the immune status of MM and for gauging the effectiveness of immunotherapy.

### 3.7 Constructing a predictive nomogram to assess the clinical applicability

**FIGURE 6:**
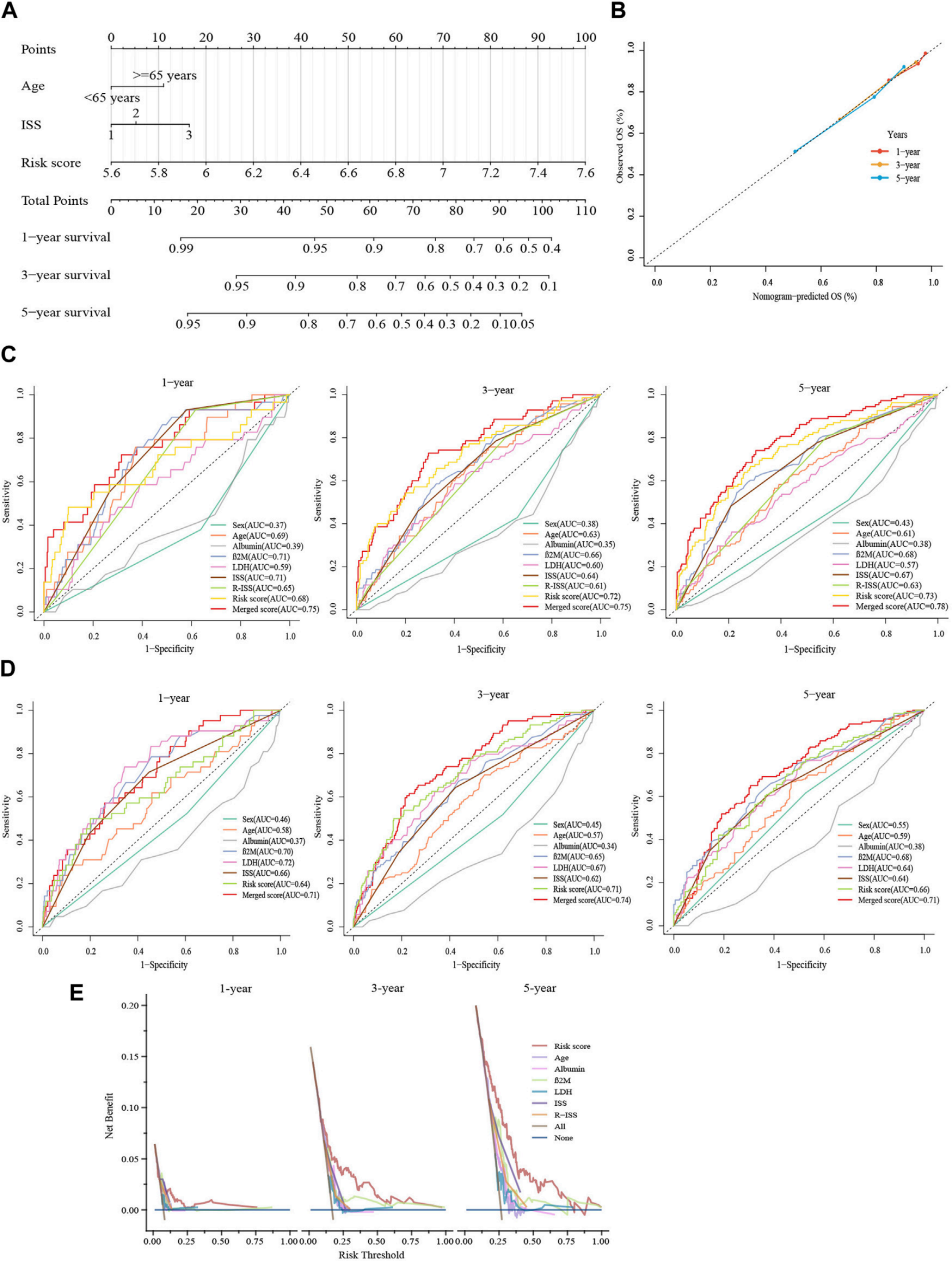
Constructing a predictive nomogram to assess clinical applicability. **(A)** The nomogram based on age, ISS phase, and glycolytic risk score in the training cohort. **(B)** Calibration plots were made to validate the accuracy in predicting 1-, 3- and 5-year survivals. **(C,D)** Time-dependent ROC analyses at 1-, 3- and 5-year with the merged score and other clinical covariates. **(E)** Survival net benefits of each clinical feature and the risk score were estimated with decision curve analysis (DCA). C shows GSE136337 (*n* = 415); D displays GSE24080 (*n* = 556).

For improving the survival prediction efficiency of the model, a combined nomogram model was constructed with age, ISS stage, and glycolytic risk score (GSE136337, *n* = 415) (Figure 6A). Considering the clinical information of the validation cohort, we did not include cytogenetics. The nomogram showed a strong predictive value, as shown by a C-index of 0.728. An ideal concordance between the forecast and observation was visible on the survival probability calibrations plot (Figure 6B). The 1-, 3- and 5-year AUCs were 0.75, 0.75, and 0.78, respectively, higher than the AUCs of the ISS and R-ISS (Figure 6C). The same was in the validation set GSE24080 (*n* = 556) (Figure 6D).

Moreover, graphical representations provided by DCA showed that the glycolytic risk score outperformed other metrics, such as the R-ISS, in terms of the net benefit to survival (GSE136337, *n* = 415) (1-year, threshold:0.125–0.250; 3-year, threshold:0.125–0.563; 5-year, threshold:0.125–0.750) (Figure 6E).

### 3.8 External validation using qRT-PCR

**FIGURE 7:**
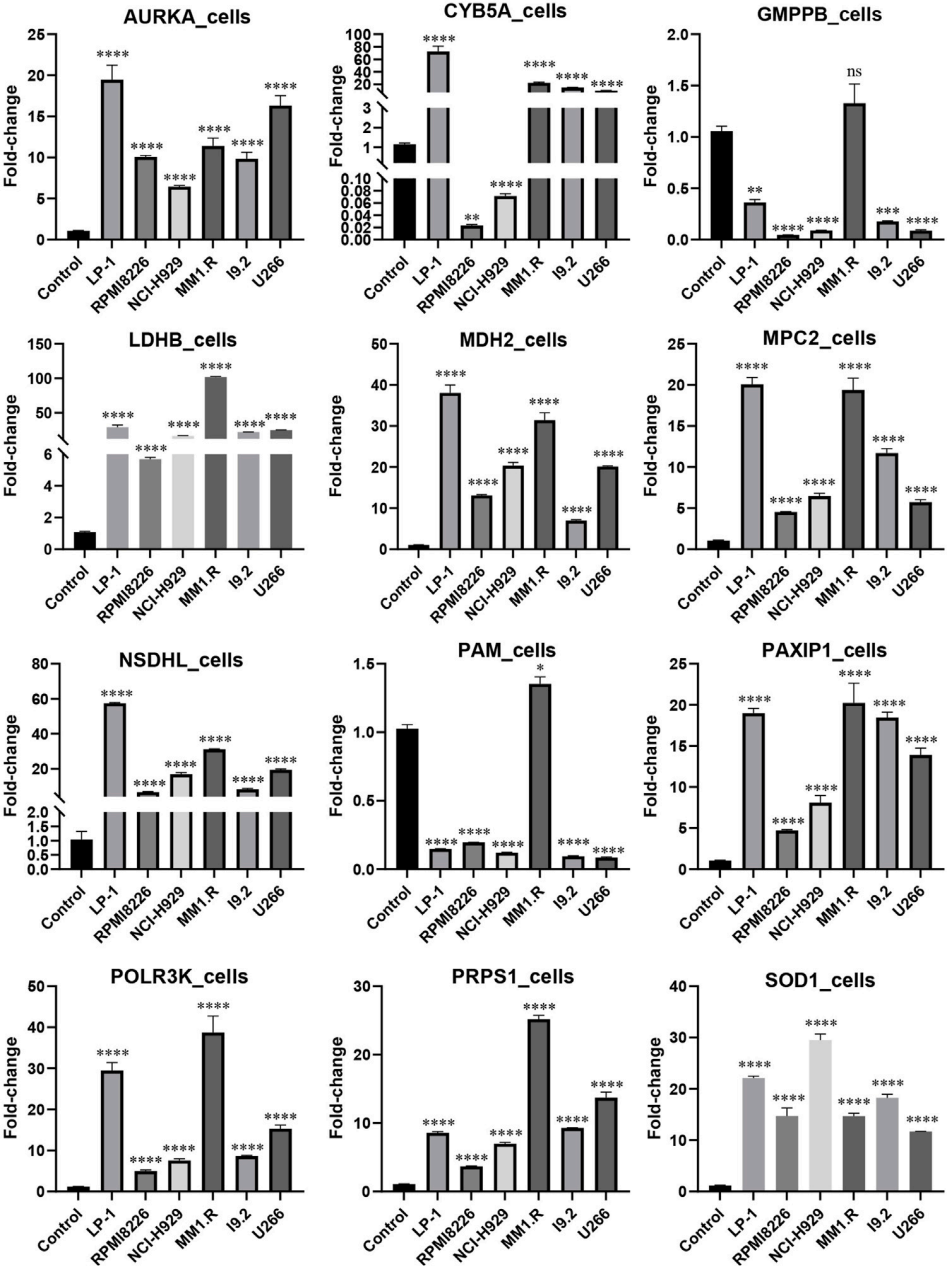
External validation in MM cell lines using qRT-PCR (Mean ± SEM). ns, no statistical significance; *p < 0.05; **p < 0.01; ***p < 0.001; ****p < 0.0001.

**FIGURE 8:**
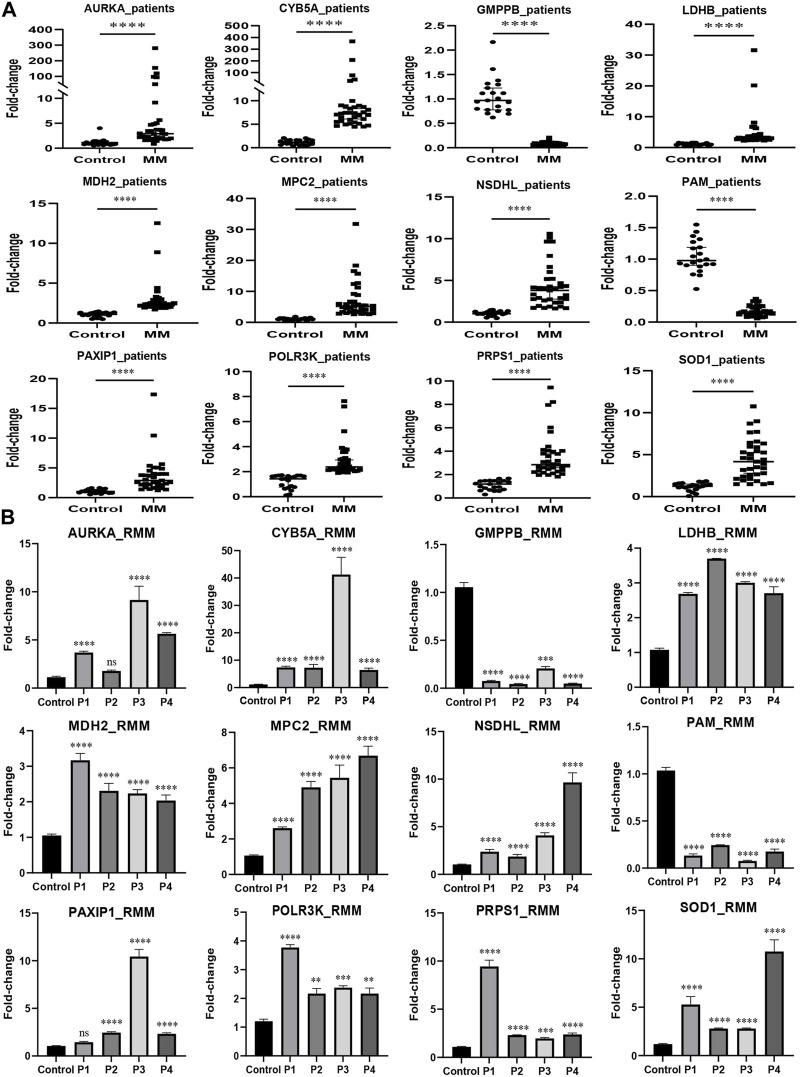
External validation in MM patients using qRT-PCR. **(A)** The expression levels of the prognostic genes in MM patients (*n* = 35). **(B)** The expression levels of the prognostic genes in RMM patients (*n* = 4) (Mean ± SEM). Control, *n* = 21; ns, no statistical significance; **p < 0.01; ***p < 0.001; ****p < 0.0001.

MM cell lines and patient samples were subjected to PCR to further validate our predictive model. In these six cell lines (LP-1, RPMI8226, NCI-H929, MM1.R, I9.2, and U266), *AURKA*, *CYB5A*, *LDHB*, *MDH2*, *MPC2*, *NSDHL*, *PAXIP1*, *POLR3K*, *PRPS1*, and *SOD1* exhibited primarily upregulation (*p* < 0.01). *GMPPB* and *PAM*, on the other hand, underwent downregulation (*p* < 0.05) (Figure 7). Correspondingly, the experimental results from the patients were consistent with the cell lines (*p* < 0.0001) (Figure 8A). We then further investigated the changes in gene expression profiles related to glycolysis among the RMM patients. Figure 8B showed that the expression of *GMPPB* and *PAM* was significantly lower than that of controls (*p* < 0.001), while the opposite was true for other genes (*p* < 0.01).

## 4 Discussion

Metabolic reprogramming, as a defining feature of cancer, has been extensively studied in recent years (Hanahan, 2022). Warburg effect is a manifestation of glucose metabolism reprogramming and is considered an adaptive mechanism to support the biosynthetic demands of uncontrolled proliferation of cancer cells (Warburg, 1956). MM is thought to be a glycolytic tumor because of an elevated glycolytic gene profile associated with disease progression (Evans et al., 2022; Misund et al., 2022) and its sensitivity to glycolysis inhibitors, such as GLUTs and key enzymes (D'Souza and Bhattacharya, 2019; Okabe et al., 2022; Yao et al., 2023). Some key enzymes in the glycolysis process are over-expressed in MM, and more highly expressed in relapsed and refractory patients, such as HK2 and LDHA (Mulligan et al., 2007; Maiso et al., 2015). At the same time, glycolysis as a new therapeutic target has attracted extensive attention. Hypoxia-inducible HK2 has been shown to induce an anti-apoptotic phenotype in myeloma cells through autophagy activation (Ikeda et al., 2020). The combination of phloretin (GLUT1 inhibitor) and daunorubicin (a chemotherapeutic drug) enhances the effect of the latter under hypoxia (Cao et al., 2007; Zub et al., 2015). The production of large amounts of lactate in glycolysis leads to acidification of the tumor microenvironment, which in turn impairs T cells’ proliferation and damages NK cells’ function. Buffering the pH can improve the efficacy of immunotherapy and can be useful in MM (Beckermann et al., 2017; Kouidhi et al., 2018; Wu et al., 2020).

Recently, advances in genomic technology have led to a better understanding of the underlying genetic abnormalities in myeloma. Gene expression profiles (GEP) are the most important tumor-related prognostic biomarkers (Chng et al., 2014). In recent years, GEP has been extensively studied as a potential tool to assess the risk of MM, and several GEP classifiers have been developed (Shaughnessy et al., 2007; Kuiper et al., 2012; Kuiper et al., 2015). However, research on biomarkers of glycolysis-related genes in MM is still limited.

In our study, we constructed a prognostic model featuring 12 GRGs using LASSO regression analysis and validated it in two independent external cohorts. The high-risk group was identified as having poor prognoses. The prognostic accuracy of the model was further assessed by the time-ROC analysis. Furthermore, the multivariate Cox analysis demonstrated that the glycolytic model was an independent prognostic factor and truly served as a powerful prognostic signature of MM.

Clinical traits and drug sensitivity varied between the subtypes. The higher-scoring group with a worse prognosis had more high-risk factors, such as higher LDH levels, ISS, RISS staging, and higher-risk cytogenetic markers. Most of the risk genes in the model were upregulated in myeloma patients, and this upregulation tendency progressed as MM developed. Furthermore, the high-risk cohort exhibited resistance to bortezomib, doxorubicin, and cytarabine. These results were consistent with previous analyses of prognosis. We found that the high-risk subgroup exhibited more responsiveness to vorinostat. As a histone deacetylases (HDAC) inhibitor, vorinostat causes cell cycle arrest and cell death, lowers angiogenesis, and modifies immunological responses in cancer cells (McClure et al., 2018). Several clinical trials have demonstrated the enormous promise of vorinostat in the combination treatment of MM (Richardson et al., 2008; Badros et al., 2009; Weber et al., 2012; Vesole et al., 2015). Chemosensitization allows it to increase the efficiency with which IMiDs or conventional chemotherapeutic drugs kill MM cells (Mitsiades et al., 2004). Panobinostat, also an HDAC inhibitor, has been approved by the U.S. Food and Drug Administration for the treatment of relapsed and refractory multiple myeloma.

Furthermore, we used the WGCNA and GSEA to explore the underlying mechanism of glycolytic prognostic gene markers. Functional analysis revealed many pathways enriched in the high-risk subgroup, including those closely related to metabolism and tumor progression. These findings suggested the strong link between poor prognosis in high-risk cohort and activation of tumor metabolic reprogramming, providing a potential molecular mechanism to elucidate the relationship between the signature and MM progression.

More and more data point to the important role of the evolution of tumor cells and their surroundings in the emergence of MM. Immune evasion and the progression of MM are made possible by the TME. Signature components such as BMSC in TME reduce immune surveillance and promote migration, proliferation, and drug resistance of malignant plasma cells (Garcia-Ortiz et al., 2021; Holthof et al., 2021). The further factors in MM cells avoiding cytotoxic T lymphocyte killing are defective antigen presentation and immunoreactive T cells during bone marrow tumorigenesis (Cohen et al., 2020; Dhatchinamoorthy et al., 2021; Samur et al., 2021). Moreover, immune escape and immunotherapy resistance have increasingly been linked to mutations in genes relevant to antigen presentation (Lee et al., 2020; Jhunjhunwala et al., 2021). And it is believed that one of the crucial mechanisms for the invasion and metastasis of cancer cells is detective intercellular adhesion (Cavallaro and Christofori, 2004; Sousa et al., 2019). In our study, the high-risk group displayed dysregulation of immune function. It had a lesser number of immune cells and more stromal cell infiltration. Additionally, a higher rejection of T cells was noted. The expression of genes related to antigen presentation and cell adhesion was negatively correlated with most risk genes, whereas the opposite was true for the protective gene *GMPPB*. Biological processes similar to those present in stem cells were also more prevalent in the high-scoring subtype. Furthermore, the high-risk group had a higher degree of tumor dedifferentiation and a subsequent higher likelihood of metastatic and recurrence (Sampieri and Fodde, 2012; Hsu et al., 2018). All of these findings point to the promise of glycolysis-related models in characterizing the immune microenvironment, predicting responsiveness to immune-targeted therapies, and assessing prognosis.

The host factors, tumor-related factors, tumor stage are considered as crucial predictors of survival outcomes by the International Myeloma Working Group (IMWG). The most significant host factor is age, the most significant tumor-related factors are genetic aberration and GEP (Chng et al., 2014). Our glycolysis-related gene signature risk model belongs to tumor-related prognostic factors. MM is highly heterogeneous, and the prognosis of patients varies greatly among individuals. A single prognostic biomarker does not meet the requirements for accurate prognosis prediction. Therefore, combining the information from the training and validation datasets, we integrated multiple prognostic factors to construct a nomogram, including age, risk score, and ISS stage. The nomogram’s ability to predict survival at various time points was the most reliable and potent of any other single variable.

In our prognostic model, *AURKA, SOD1*, *MPC2*, *LDHB*, *PAXIP1*, *MDH2*, *PRPS1*, *CYB5A*, *POLR3K*, and *NSDHL* were identified as risk genes, while *PAM* and *GMPPB* were identified as protective genes. The majority of them are reportedly closely related to the occurrence and progression of cancer. *AURKA* is a serine/threonine kinase that regulates chromosome arrangement, centrosome amplification, and mitotic spindle formation (Hirota et al., 2003; Fu et al., 2007). Overexpression of *AURKA* has been linked to oncogenic transformation, including chromosomal instability and disruption of multiple oncoprotein regulatory pathways and tumor suppressors (Zhou et al., 1998; Lens et al., 2010; Nikonova et al., 2013). Through *AURKA*-mediated phosphorylation of *LDHB*, glycolysis and biosynthesis were effectively promoted, thereby promoting tumor progression (Cheng et al., 2019). *AURKA* participates in the control of NF-κB and Wnt/β-catenin pathways, associated with the resistance and progression of MM (Dutta-Simmons et al., 2009; Mazzera et al., 2013; Zhou et al., 2013; Huynh et al., 2018). Both *in vitro* and *in vivo*, inhibition of *AURKA* can induce apoptosis and cell death of MM (Shi et al., 2007; Gorgun et al., 2010). *AURKA* inhibitor can synergize with BTZ to kill t (4,14)-positive MM cells (Jiang et al., 2022). Several *AURKA* inhibitors are currently being studied in clinical trials in MM or other cancers (Dees et al., 2012; Caputo et al., 2014; Kelly et al., 2014; Long et al., 2015). In the phase I clinical trial studies of RMM, *AURKA* had shown potential efficacy (Gorgun et al., 2010). Superoxide dismutase (*SOD*) catalyzes the disproportionation of superoxide free radicals into hydrogen peroxide and molecular oxygen. *SOD1* (CuZnSOD) is the main *SOD* in mammals (Crapo et al., 1992; Tsang et al., 2014). *SOD1* overexpression is associated with MM progression and poor prognosis, as well as bortezomib resistance (Salem et al., 2015; Wang et al., 2020; Du et al., 2021). Dysregulation of the intrinsic oxidative environment is an important feature of cancer cells, including MM cells (Aykin-Burns et al., 2009). As an antioxidant, *SOD1* had been shown to counteract the cytotoxic effects induced by proteasome inhibitors, which relied mainly on the production of ROS (McConkey and Zhu, 2008; Salem et al., 2015). *M*PC2 and *LDHB* were found to be associated with the carfilzomib-related cardiotoxicity in MM (Tantawy et al., 2021). And they were linked to the reorganization of BTZ metabolism in MM cells, which results in resistance to BTZ and poor prognosis (Findlay et al., 2023). *MPC2* is one of the rate-limiting proteins involved in glycolytic metabolism. Inhibition of *MPC* complexes impaired myeloma cell core bioenergetics to increase proteasome inhibitor-induced anti-MM effects (Findlay et al., 2023). Silencing *LDHB* selectively inhibited basal autophagy and cell proliferation in cancer cells and induced cell death (Brisson et al., 2016). For humoral immunity (Su et al., 2017) and the reorganization of B lymphocyte class switches (Daniel et al., 2010), *PAXIP1* is necessary. It is reported that *PAXIP1* was necessary to encourage osteoclast differentiation to maintain bone marrow niche structure (Das et al., 2018). Upregulation of *PAXIP1* promoted cell proliferation and inhibited apoptosis (Ma and Zheng, 2021). *MDH2* is one of the major players in the malate-aspartate shuttle (Minarik et al., 2002). Through increased cell viability and reduced apoptosis, *MDH2* contributed to the doxorubicin resistance (Lo et al., 2015). The above may explain the resistance to bortezomib and doxorubicin in the high glycolysis scoring group. *PRPS1* is a crucial enzyme in nucleotide synthesis. Mutations in *PRPS* increase the metabolic vulnerability of the patients with acute lymphoblastic leukemia (ALL), thereby reducing relapse and progression (Srivastava et al., 2021; Song et al., 2023), which is also seen in colorectal and hepatocellular carcinomas (Li et al., 2016; Jing et al., 2019). As a membrane-bound cytochrome, *CYB5A* carries electron for several membrane-bound oxygenases (Hegesh et al., 1986; Kurian et al., 2006). Studies have shown that *CYB5A* is upregulated in recurrent ALL (Bartsch et al., 2022). *POLR3K* is responsible for synthesizing transfer and small ribosomal RNAs in eukaryotes. *POLR3K* may contribute to the proliferation and angiogenesis of cancer cells by inducing NF-B signaling. (Ablasser et al., 2009; Chiu et al., 2009; Taniguchi and Karin, 2018). The cholesterol-metabolizing enzyme *NSDHL* is a potential metastatic driver in breast cancer (Yoon et al., 2020; Chen et al., 2021). *PAM* plays an auxiliary role in amidation and regulates the activity of peptides in the adrenal medulla and pheochromocytoma cells. Oligonucleotide hybridization and immunohistochemical staining showed that *PAM* was downregulated in neuroendocrine tumors, which was associated with the malignant behavior of the tumors (Thouennon et al., 2007; Horton et al., 2020). *GMPPB* was negatively associated with poor outcomes in endometrial cancer (Wang et al., 2019; Liu et al., 2020). The relationship between some genes and MM still needs further study.

However, our study has several limitations that need to be addressed. First, the construction and validation of our model relied on the retrospective data from the public database and our clinical samples, so the prognostic robustness and clinical utility of the glycolysis-associated gene signature need to be further verified in larger prospective studies. Secondly, the validation datasets we used lacked complete clinical information, such as detailed cytogenetic information. Finally, the specific role of each gene in MM is unclear, and additional studies *in vivo* and *in vitro* are further needed.

## 5 Conclusion

In summary, our research offers a fresh perspective for comprehending glycolysis’ function in MM. Different glycolysis-related patterns exhibited heterogeneity in terms of clinical traits and the sensitivity of chemotherapeutic drugs and immunotherapy. This prognostic signature was highly coordinated with multiple malignant features such as immune dysfunction, stem cell-like features, and cancer-related pathways, and was associated with survival outcomes in MM patients. Its clinical effectiveness had been further validated, showing promise in prognostic assessment and treatment options for MM patients.

## Figures and Tables

**Table:** 

**MM**	Multiple myeloma
**MGUS**	monoclonal gammopathy of undetermined significance
**SMM**	smoldering myeloma
**BTZ**	bortezomib
**IMiD**	immunomodulatory drug
**R-ISS**	The Revised International Staging System
**CAs**	cytogenetic abnormalities
**FISH**	fluorescent *in situ* hybridization
**LDH**	lactate dehydrogenase
**GRGs**	glycolysis-related genes
**NDMM**	newly diagnosed multiple myeloma
**GLUTs**	glucose transporter inhibitors
**HK2**	hexokinase 2
**3-BP**	3-bromopyruvate
**qRT-PCR**	quantitative real-time PCR
**GEO**	Gene Expression Omnibus
**GSEA**	Gene Set Enrichment Analysis
**LASSO**	Least absolute shrinkage and selection operator
**PPI**	protein-protein interaction
**TCGA**	The Cancer Genome Atlas
**CCLE**	Cancer Cell Line Encyclopedia database
**WGCNA**	weighted gene co-expression network analysis
**GO**	Gene Ontology
**KEGG**	Kyoto Encyclopedia of Genes and Genomes
**TME**	tumor microenvironment
**ssGSEA**	the single-sample gene set enrichment analysis
**ICI**	immune checkpoint inhibitor
**TIDE**	tumor immune dysfunction and exclusion
**IPS**	immunophenotype score
**MHC**	antigen presentation
**EC**	effector cells
**SC**	suppressor cells
**CP**	checkpoint markers
**time-ROC**	time-dependent receiver operating characteristic
**DCA**	decision curve analysis
**NB**	net benefit
**NRMM**	non-relapsed MM
**RMM**	relapsed MM
**ANOVA**	one-way analysis of variance
**HRCAs**	high-risk cytogenetic abnormalities
**SNPs**	single nucleotide polymorphisms
**SNVs**	single nucleotide variants
**CNVs**	copy number variations
**BMSC**	bone marrow stromal cells
**GEP**	gene expression profiles
**HDAC**	histone deacetylases
**IMWG**	International Myeloma Working Group
**ALL**	acute lymphoblastic leukemia

## Data Availability

The datasets presented in this study can be found in online repositories. The names of the repository/repositories and accession number(s) can be found in the article/Supplementary Material.

## Supplementary material

SUPPLEMENTARY FIGURE S1 Validation of the prognostic model. **(A,B)** Kaplan-Meier curves for the validation datasets between the high- and low-scoring subtypes. **(C,D)** The disparities in the 12 genes’ expressions and survival outcomes. **(E,F)** Time-dependent ROC analysis was used to evaluate the model’s sensitivity and specificity. **(A,C,E)** display GSE24080 (*n* = 556); **(B,D,F)** display GSE4204 (*n* = 534).
