# Supplementary figures and images for: A novel glycolysis-related gene signature for predicting the prognosis of multiple myeloma
Source: Front Cell Dev Biol. 2023 Jun 2;11:1198949. doi: 10.3389/fcell.2023.1198949 (PMC10272536; doi:10.3389/fcell.2023.1198949)

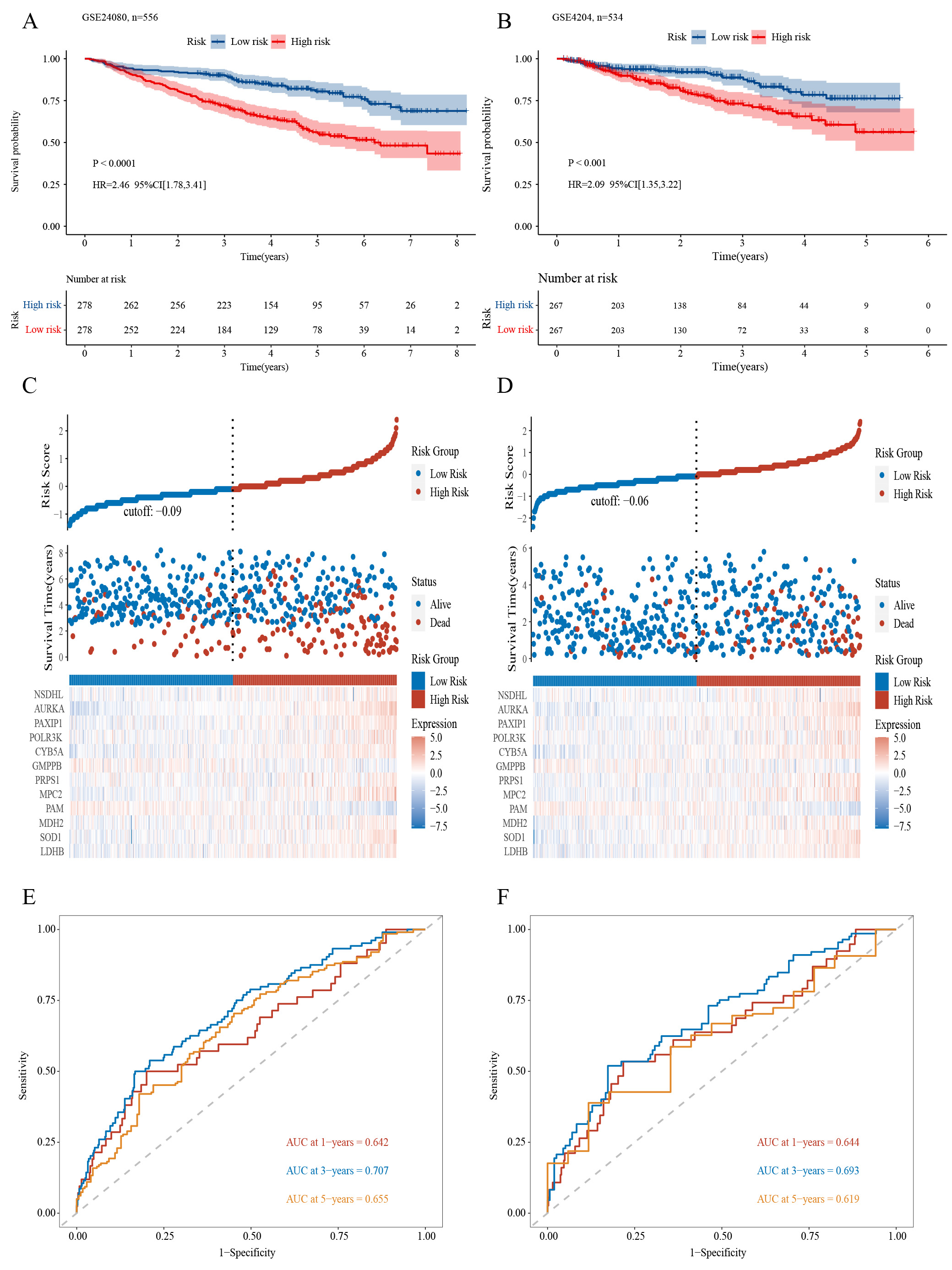

Supplement: Supplementary file 1 [file Image1.JPEG]
